# Nano-coating with silicon dioxide to reduce the occurrence of bacterial contamination in a pig abattoir drinking system

**DOI:** 10.1007/s12223-025-01243-x

**Published:** 2025-02-04

**Authors:** Celine Buder, Nina Langkabel, Alina Kirse, Mirjam Kalusa, Simone A. Fietz, Diana Meemken

**Affiliations:** 1 Institute of Food Safety and Food Hygiene, Working Group Meat Hygiene, School of Veterinary Medicine, Freie Universität Berlin, 14163 Berlin, Germany; 2 Veterinary Centre for Resistance Research, School of Veterinary Medicine, Freie Universität Berlin, 14163 Berlin, Germany; 3 Institute of Biometry, Epidemiology and Information Processing, WHO Collaborating Centre for Research and Training for Health at the Human-Animal-Environment Interface, University of Veterinary Medicine Hannover, 30559 Hannover, Germany; 4 Institute of Veterinary Anatomy, Histology and Embryology, Faculty of Veterinary Medicine, University of Leipzig, 04103 Leipzig, Germany

**Keywords:** Nano-coating, Silicon dioxide, Biofilm, Swine, Slaughterhouse, Drinking water distribution system

## Abstract

A recently discovered source for infection of slaughter pigs, and thus entry for bacteria into the food chain, is the installed drinking equipment in lairage pens of pig abattoirs. To mitigate this, nano-coating of stainless steel, currently used in human medicine fields as well as in other parts of the food chain, appears as promising technology. In this study, silicon dioxide nano-coating was applied to six drinkers and installed for one and three months in a lairage of a pig abattoir, while results were compared with those of drinkers that had not been nano-coated. Laboratory examination of eight sample types related to the drinkers was conducted for total aerobic plate count, *Enterobacteriaceae* count, *Pseudomonas* spp. count, *Salmonella* presence, pathogenic *Yersinia enterocolitica* presence, *Listeria monocytogenes* presence and methicillin-resistant *Staphylococcus aureus* presence. The nipple drinker, which the pigs take into their mouth for drinking, was then examined using scanning electron microscopy and elemental analysis. The nano-coating did not produce statistically significant reductions in the loads or presence of these bacteria compared to the same but uncoated drinking equipment used under the same conditions. Further studies should focus on the implementation of combined methods, such as nano-coating and sanitary treatment, as well as modifications to the coating itself, to produce meaningful reductions of the bacterial loads on/in abattoir lairage drinking equipment.

## Introduction

A nowadays widely known and used method to reduce the development of bacterial biofilms is based on nanotechnology (Ferreira et al. 2010). It is especially common in the medical sector (Ramasamy and Lee 2016) and well developed on medical devices (Lukowski et al. 2019). Next to the high value of nanotechnology in the medical sector, a more recent development is the use of nanotechnology in food production equipment (Hamad et al. 2018). Recent studies concentrated on nanotechnology use close to the food itself (Cruz-Lopes et al. 2021), and even though nano-sized food additives have been used since the middle of the twentieth century, the methodologies to use nanoparticles are still increasing (Winkler et al. 2016). Recent findings portray the possibility of using different nano-sized particles in food, including the advantages and disadvantages (Su et al. 2022). An already known and approved use of nanotechnology in the food industry is the coating of different processing and production surfaces at risk of contamination or biofilm formation (Sekhon 2010; Türetgen 2015). These coatings can aim for different effects, i.e. antifouling (Banerjee et al. 2011), antiadhesive (Neoh and Kang 2011) or direct/indirect antimicrobial characteristics (Shintani 2004). Possible materials used for these coatings differ depending on whether an antimicrobial or an antiadhesive effect is preferred (Ji and Zhang 2009). Silicon dioxide nano-material primarily reduces the attachment capability of microorganisms (Sami et al. 2021), while other materials, such as nano-metals, are able to reduce microorganisms directly due to elemental characteristics, i.e. silver (Amirsoleimani et al. 2018; Ivanova et al. 2011), gold (Díaz et al. 2007) or titanium (Maddikeri et al. 2008; Neoh et al. 2012; Puckett et al. 2010). Generally, nano-sized coatings are able to reduce both the attachment of microorganisms and bacterial proliferation and, thus, reduce biofilm formation as well (Banerjee et al. 2019). Blaeske et al. (2023) were able to show the reduced adherence of *Campylobacter jejuni* on silicon dioxide-coated stainless steel in a laboratory environment after inoculation, while a study by Schumann-Muck et al. (2023) reported a slight decrease of *Salmonella* and *Escherichia coli* on silicon dioxide-coated rubber picker fingers—a poultry slaughter device used for defeathering—after inoculation.

To further investigate the impact of nanotechnology in the food industry, this study focused on the newly discovered source of bacterial contamination through the drinking equipment installed in pig abattoir lairage pens (Buder et al. 2023). The drinking equipment can simultaneously act as a transmitting device whereby pigs can introduce specific pathogens into the abattoir environment, and as a result, can be infected. Pig infections are possible in the lairage environment, as a study by Hurd et al. (2001) described pig infection with *Salmonella* (*S.*) Typhimurium after the animals were exposed to contaminated lairage pens for just 2 h. Therefore, the aims of this study were to examine silicon dioxide nano-coated drinking equipment (NCDE) and to evaluate the potential effect in comparison to uncoated drinking equipment (UCDE), investigated previously (Buder et al. 2023), with respect to reductions of biofilms and specific pathogens in the same lairage pens. Multiple sample types were investigated from different risk-based positions in the drinking equipment. The samples studied were drinker water samples to evaluate the bacterial contamination of the ingested water, the stainless-steel surfaces in direct contact with the pigs’ mouths to examine the surfaces’ superficial hygienic status, and the inner surfaces of the drinking equipment without direct contact with the pigs to detect retrograde microbial growth.

## Materials and methods

In this investigation, the same commercial German pig abattoir and the exact same lairage pens were selected to ensure comparability with the previously conducted study regarding the general occurrence of biofilm and specific pathogens in the drinking equipment (Buder et al. 2023). This comparability necessarily included using the same sample types, techniques and laboratory methods.

### Drinker selection, material and surface coating

**Fig. 1:**
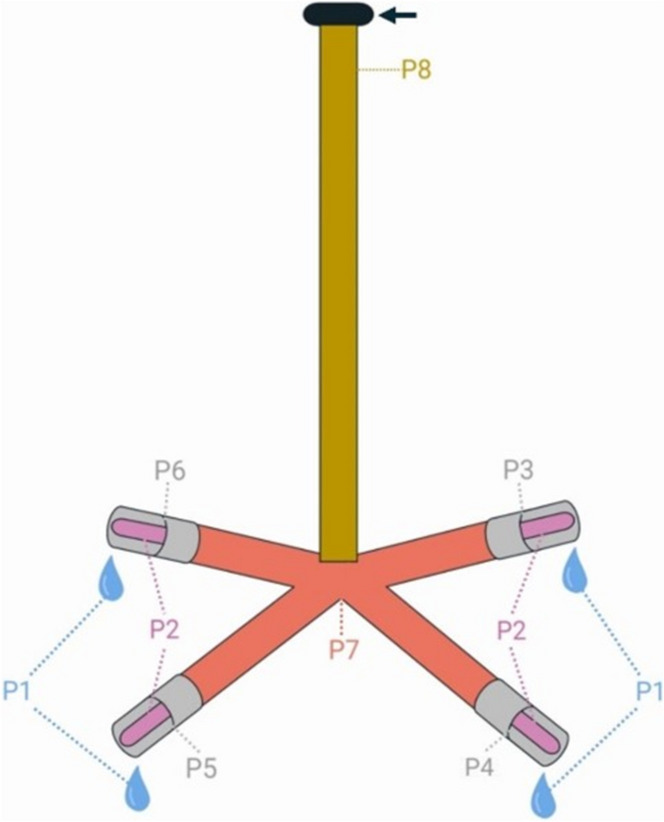
Schematic overview of the drinker, the sample types and the connection to the main water distribution system (arrow); P1—water sample; P2—pooled nipple swab sample from the outside; P3 to P6—individual sample of one complete nipple; P7—pooled lower pipe section swab sample from the inside; P8—pooled upper pipe section swab sample from the inside

Six out of the thirty lairage pens (as in Buder et al. (2023)) were selected throughout the lairage of the abattoir to represent a homogenous allocation regarding occupancy (17 to 19 pigs) and size, as well as heterogenous details in terms of distance to the source of water (impacting water velocity and pressure), accessibility of the pens and occurring dead ends (resulting in a higher possibility of still, standing water). Each selected pen held one drinker, named Drinker A to F for the six lairage pens. The main water distribution system connecting all pens was made of stainless steel, and the link to each drinker consisted of flexible elements of rubber, similar to materials used for domestic garden hoses. The drinkers, also made of stainless steel, consisted of an 88 cm long pipe, ending in a four-fold pipe cross, each end finishing with a nipple drinker, resulting in four nipple drinkers per drinker (Fig. 1). The water consisted of 75% artesian water from a well owned by the abattoir and 25% tap water from the public water supply system. It was tested regularly by the abattoir, in accordance with legal German Drinking Water Regulation (TrinkwV 2023), and met the water quality standards.

Drinking equipment was commercially supplied (Hölscher & Leuschner, Emsbüren, Germany) and commercially coated with a nano-sized layer based on silicon dioxide. The polymerised silicon dioxide molecules form a continuous layer, due to self-organisation, of approximately 50–80 nm, which is tightly linked to the stainless-steel surface of the drinking equipment. To achieve an evenly applied coating on the complex elements, the drinkers were immersed into the coating solution as a whole. After the drinkers were immersed, a dripping and drying process followed, which is crucial for the adhesion and durability of the coating. To ensure the end product was of suitable quality, the selection of the coating material, the adjustment of the dripping speed and duration and the control of the process parameters were previously optimised and were conducted in the same way for all drinkers.

### Sample types

Eight sample types were taken per drinker (Fig. 1). Firstly, a pool sample of water was obtained from all four nipple drinkers by releasing each nipple drinker individually with newly gloved hands. From each nipple drinker, approximately 25 mL of water were filled into one empty, sterile, plastic tube (Sarstedt AG & Co. KG, Nümbrecht, Germany), in the following described as P1. Next, the drinkers were dismantled from the main water distribution system. All following swab samples were taken with dry and individually sterilised swabs (Sarstedt AG & Co. KG, Nümbrecht, Germany) and broken off to ensure only parts of the swab sticks that had not been in contact with a gloved hand remained in the empty, sterile, plastic sample tubes. Sample type P2 consisted of a pooled swab sample from all four nipple drinkers per drinker. Each nipple drinker was sampled with two swabs. The first was used to swab the still-wet nipple drinker, while the second swab was used to swab the then dried area. Only the frontal part of the nipple drinker was sampled for this sample type, which included the area where water emerges, and with which the pigs have direct contact. All eight swabs per drinker were put into one sample tube. Thereafter, the four nipple drinkers were disassembled and placed separately in sample tubes (P3, P4, P5 and P6). Sample type P7 consisted of another pooled sample of eight swabs, which were used to sample the inner surface of the four-fold pipe cross. Circular movements from the pipe cross to each opening were used to cover the whole area of 7 cm length. This was repeated with two swabs in sequence for all four parts of the pipe cross. All eight swabs were put in a sample tube, as described above. The last sample type (P8) tested the upper pipe section, where the drinker was connected to the main water distribution system. Two swabs were used to sample the inner surface of the pipe, one after the other, in circular movements from the top end of the drinker for the length of the swab. The two swabs per drinker were again placed into one sample tube.

The first set of new, commercial, nano-coated drinkers were installed in the selected lairage pens for one month and then sampled (samples P1 to P8, see above). Afterwards, another set of new, commercial, nano-coated drinkers were installed. After three months in use, the second sampling (P1 to P8) was conducted.

### Bacterial examination

The samples were sent immediately after collection in a cooled box at 6–10 °C to the laboratory of the Institute of Food Safety and Food Hygiene, Working Group Meat Hygiene at Freie Universität Berlin, Germany. Once they arrived the next morning, the examination started in accordance with the respective German standards (DINs): quantitative analysis of total aerobic plate count (APC) (DIN 10161:^2016^–12), *Enterobacteriaceae* (EB) count (DIN 10164–2:^2019^–06), *Pseudomonas* spp. count (DIN EN ISO 13720:^2010^–12), and qualitative analysis of *Salmonella* (DIN EN ISO 6579–1:^2020^–08), pathogenic *Yersinia (Y.) enterocolitica* (DIN EN ISO 10273:^2017^–08), *Listeria (L.) monocytogenes* (DIN EN ISO 11290–1:^2017^–09) and methicillin-resistant *Staphylococcus aureus* (MRSA) (DIN EN ISO 6888–1:^2022^–06).

All sample types were tested for APC and EB, and sample types P2, P3–P6, P7 and P8 were tested for *Pseudomonas* spp. While P1 was directly processed following the respective standard procedures for water (BVL L 59.00–5:^1988^–05; BVL L 59.00:^2010^–01), the other sample types (P2, P3 to P6, P7 and P8) were diluted with additional 50 mL buffered peptone water (BPW; Merck KGgA, Darmstadt, Germany) and stored in the refrigerator for 30 min. Better dissolution of the noticeable particles was accomplished after homogenisation using a whirl mixer (Phoenix Instrument GmbH, Garbsen, Germany) for 30 s for sample types P2, P7 and P8 and manually for 1 min for P3 to P6. A part of each original homogenate (dilution level 0 or 10^0^) was diluted in a 1:9 ratio with sodium peptone agar (Merck KGgA, Darmstadt, Germany). Plate Count Agar (PCA; Th. Geyer GmbH & Co. KG, Renningen, Germany) was used for the examination of APC and Violet Red Bile Dextrose Agar (VRBD; Merck KGgA, Darmstadt, Germany) for examination of EB counts. For both examinations, 0.05 mL of each dilution was dropped on the respective agar surface and streaked out using a sterile loop (Sarstedt AG & Co. KG, Nümbrecht, Germany) for P1, P2, P3 to P6 and P7. For P8, 0.1 mL of each dilution was applied and spread using a sterile spatula (Sarstedt AG & Co. KG, Nümbrecht, Germany). All samples were then incubated for 72 ± 2 h at 30 °C (APC) (DIN 10161:^2016^–12) or for 48 h at 37 °C (EB) (DIN 10164–2:^2019^–06). For the examination of *Pseudomonas* spp. using *Pseudomonas* CFC Agar (Oxoid Deutschland GmbH, Wesel, Germany), 0.1 mL of dilution levels 0 and 1 was spread with a sterile spatula and incubated for 40–48 h at 25 °C (DIN EN ISO 13720:^2010^–12). For the quantitative analysis of APC, EB and *Pseudomonas* spp., all colony forming units (CFU) were counted per sector on the respective agars of two consecutive dilution levels. A weighted average was obtained before being directly transformed to the logarithm base of 10, which in the case of P1, was per mL. Samples P2, P7 and P8 were calculated per cm^2^, and therefore, the surface area swabbed had to be included: P2 = 88.5 cm^2^, P7 = 149.5 cm^2^, P8 = 70 cm^2^. P3 to P6, the nipple drinkers, had an unknown surface area due to the complexity of these structures. Based on the respective German standards (DIN 10113–1:^2023^–02; DIN EN ISO 18593:^2018^–10), the calculation was conducted with the area of 1, resulting in the bacteria being counted as CFU per nipple drinker.

Sample types P2, P3 to P6, P7 and P8 were also examined for the occurrence of the specific pathogens. For the examination of *Salmonella*, the remaining part of each original homogenate, left after parts were used for other tests, was incubated as a pre-enrichment for 16–18 h at 37 °C. Afterwards, 1.1 mL were transferred into two enrichment broths, i.e. 0.1 mL into Rappaport–Vassiliadis-Soya broth (Merck KGaA, Darmstadt, Germany) incubated for 24 h at 42 °C, and 1 mL into Muller-Kauffmann Tetrathionate Novobiocin broth (Merck KGaA, Darmstadt, Germany) incubated for 24 h at 37 °C. The next day, 0.01 mL was spread with sterile loops onto two agars, Brilliant Green Agar (Oxoid Deutschland GmbH, Wesel, Germany) and Rambach-Agar (VWR International GmbH, Dresden, Germany), before incubation for 24 h at 37 °C. To examine for the occurrence of pathogenic *Y. enterocolitica*, 5 mL of each original homogenate were added to a specific pre-enrichment broth (Peptone Sorbitol Bile Broth; Sigma-Aldrich Chemie GmbH, Steinheim, Germany) and incubated for 40–48 h at 25 °C. Additionally, 0.5 mL was spread onto *Yersinia* selective medium (CIN; Oxoid Deutschland GmbH, Wesel, Germany) and incubated for 24 h at 30 °C. Another 5 mL of each 10^0^ dilution was added into a pre-enrichment broth specifically for *L. monocytogenes* (Half Fraser Broth; Oxoid Deutschland GmbH, Wesel, Germany) and incubated for 24–26 h at 30 °C. Afterwards, 0.1 mL of the pre-enrichment was spread onto ALOA (VWR International GmbH, Dresden Germany) and Palcam Agar (Oxoid Deutschland GmbH, Wesel, Germany)—two *Listeria* selective agars—and incubated for 48 h at 37 °C. In addition, 0.1 mL of the pre-enrichment was transferred to another enrichment broth (Fraser selective medium; Oxoid Deutschland GmbH, Wesel, Germany) and incubated for 24 ± 2 h at 37 °C. Finally, for MRSA examination, 0.1 mL of each original homogenate was applied onto Baird Parker Agar (Oxoid Deutschland GmbH, Wesel, Germany) and Columbia CNA Agar (Oxoid Ltd., Basingstoke, UK), spread with a sterile spatula and incubated for 24 h at 37 °C. Further procedures regarding the examination of the specific pathogens were performed according to the respective DINs (DIN EN ISO 6579–1:^2020^–08; DIN EN ISO 6888–1:^2022^–06; DIN EN ISO 10273:^2017^–08; DIN EN ISO 11290–1:^2017^–09). For serotyping the identified *Salmonella*, the Kauffmann-White-Le Minor scheme was used (Grimont and Weill 2007). Identification of other confirmed specific pathogens was performed by specific polymerase chain reactions (PCR), for pathogenic *Y. enterocolitica* according to Garzetti et al. (2014), for *L. monocytogenes* according to Bubert et al. (1999), and for MRSA according to Strommenger et al. (2003) and Jonas et al. (2002).

The results are presented by sampling (nano-coated drinking equipment installed for one (NCDE1) and three (NCDE3) months and for uncoated drinking equipment installed for one (UCDE1) and three (UCDE3) months) and sample type for every tested bacterial count or qualitative analysis for a specific microorganism. While the sample types P1, P2, P7 and P8 are separate values, sample types P3 to P6 must be evaluated as a mean count per nipple for the whole drinker, because the four nipple drinkers installed per drinker cannot be precisely allocated to any one part of the four-fold pipe cross.

### Scanning electron microscopy (SEM) and energy dispersive X-ray (EDX) analysis

One representative coated and one representative uncoated nipple, which had been in use for three months, were analysed by SEM–EDX analysis in order to compare the surface properties and compositions. Each nipple drinker was dismantled into the cone and the nipple, and the isolated nipple was used in the analysis.

**Fig. 2:**
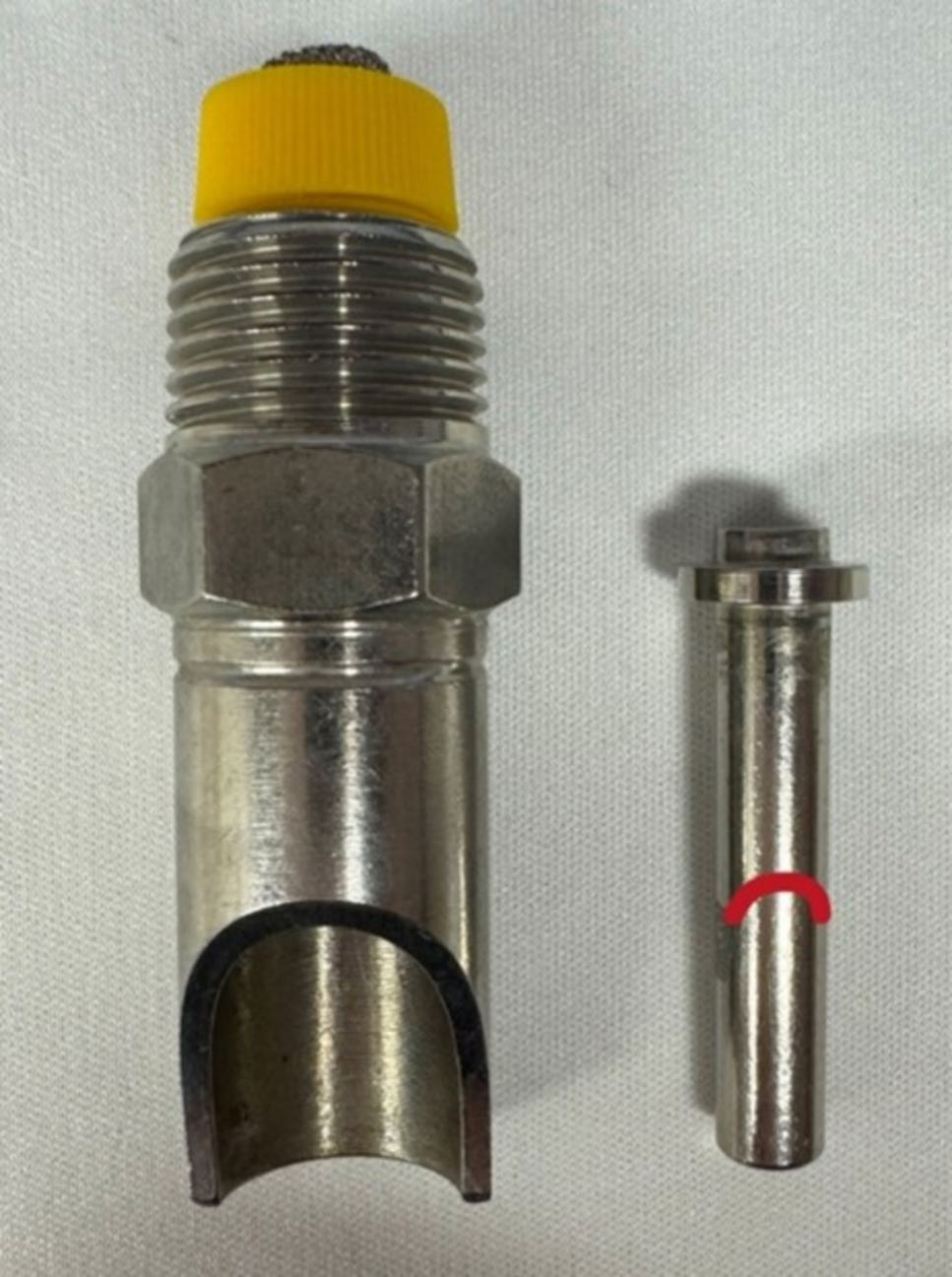
Example of a disassembled nipple drinker viewed from the front as used in this study. The pressure cone (left) was removed from the nipple (right), and the isolated nipple was used in the SEM–EDX analysis. The red line on the nipple marks the border between the posterior and the anterior region of the nipple, with only the former being completely surrounded by the pressure cone before dismantling

The posterior region on the nipple was defined as that part near the screw head that was completely surrounded by the cone before disassembly and, therefore, was inaccessible to the pigs’ teeth. The anterior region on the nipple was defined as that which was not enclosed by the cone and so was able to be bitten by the pigs’ teeth (Fig. 2). A notch in the stainless steel was defined as a morphological indentation with a minimum depth of 50 µm.

From each nipple drinker, a total of 30 point SEM–EDX analyses were carried out; 10 on a posterior position (on a smooth surface), 20 on an anterior position (on a smooth surface, *n* = 10; within a notch, *n* = 10). SEM–EDX analyses were carried out using a Zeiss EVO LS 15 scanning electron microscope (Zeiss, Oberkochen, Germany) equipped with a LaB6 source, an Everhart–Thornley secondary electron detector, a VPSE-G4—variable pressure secondary electron detector, a HDBSD—solid state backscattered electron detector with five quadrants and a SmartEDX EDX detector. The EDX analysis was performed with the following parameters: 300 × magnification; EHT 10 kV; I-Prob 5 nA; WD 9.50 mm; LiveTime 30 s; Amp Time 0.24 µs; Resolution 154.1 eV. The parameters for the images were as follows: EHT 20 kV; I-Prob 300 pA; WD 9.50 mm; 122X and 156X, respectively.

### Statistical analysis

For the microbiological examinations, descriptive analysis was conducted for each sampling of the installed drinking equipment in relation to the sample types and for each sample type in relation to the sampling, respectively. Outliers were not excluded from further analyses due to the low sample size and general plausibility. The Kruskal–Wallis test was performed for each sample type separately including the sampling as a fixed effect to examine mean differences (Table 2). Dwass-Steel-Critchlow-Fligner post hoc test was used afterwards for pairwise comparisons of UCDE with NCDE for both sampling periods and of the different sampling periods when NCDE was used (Table 3). Pairwise comparison for UCDE1 and UCDE3 samplings was not included in the results since this difference was already discussed in Buder et al. (2023) and is not the focus of this study. A significance level of ≤ 0.05 (*P* value) was chosen for all analyses. All statistical analyses were conducted using SAS®, version 9.4 (SAS Institute Inc., Cary, NC, USA).

For SEM–EDX analysis, statistical analysis was carried out using GraphPad Prism 8.4.3 software (GraphPad Software Inc., San Diego, USA). Normal distribution was tested by Anderson–Darling test and Shapiro–Wilk test. Since the data distribution was not normal, data were analysed by Kruskal–Wallis test followed by Dunn’s post hoc test. *P* values of ≤ 0.05 were considered statistically significant. Significance levels were split further into *P* ≤ 0.01, *P* ≤ 0.001 and *P* ≤ 0.0001.

## Results

### Individual categories of bacteria and specific pathogens on NCDE

#### APC

In NCDE installed for one month, APC in P1 ranged from 3.18 to 5.12 log_10_ CFU per mL and in P2 from 6.14 to 6.79 log_10_ CFU per cm^2^. For P3 to P6, the mean APC ranged from 7.49 to 8.40 log_10_ CFU per nipple. P7 showed APC of between 1.80 and 3.71 log_10_ CFU per cm^2^ and P8 showed up to 3.49 log_10_ CFU per cm^2^, while one drinker showed no APC bacterial growth (Table 2).

After the NCDE was installed for three months, APC levels in P1 were between 3.20 and 5.24 log_10_ CFU per mL. In P2, APC ranged from 5.73 to 7.20 log_10_ CFU per cm^2^, and the mean APC for P3 to P6 ranged from 7.09 to 8.04 log_10_ CFU per nipple. APC in P7 ranged from 2.16 to 3.83 log_10_ CFU per cm^2^, and P8 showed APCs up to 2.48 log_10_ CFU per cm^2^, with one drinker showing no APC bacterial growth (Table 2).

#### EB count

In NCDE installed for one month, two drinkers showed EB counts in P1 of 1.30 log_10_ CFU per mL. P2 samples from three out of six drinkers had EB counts from 2.05 to 3.60 log_10_ CFU per cm^2^. P3 to P6 had the highest occurrence of EB. All drinkers were positive for EB, and counts ranged from 1.86 to 4.09 log_10_ CFU per nipple. In P7 and P8, all samples were negative for EB (Table 2).

In NCDE installed for three months, no EB could be detected in P1, P7 or P8. P2 samples were positive in all six drinkers for EB, with counts ranging from 1.05 to 2.88 log_10_ CFU per cm^2^, as were P3 to P6, where counts ranged from 1.92 to 4.64 log_10_ CFU per nipple (Table 2).

#### *Pseudomonas* spp. count

*Pseudomonas* spp. was detected in all sample types of the NCDE installed for one month. The counts in P2 ranged from 2.17 to 4.19 log_10_ CFU per cm^2^ and in P3 to P6 from 3.38 to 5.59 log_10_ CFU per nipple. Two samples of P7 were positive for *Pseudomonas* spp., with counts of up to 2.19 log_10_ CFU per cm^2^. One sample of P8 also harboured this microorganism (1.93 log_10_ CFU per cm^2^) (Table 2).

In NCDE installed for three months, P2 and P3 to P6 were positive for *Pseudomonas* spp. Four samples of P2 contained up to 2.49 log_10_ CFU per cm^2^, and all P3 to P6 samples were positive, with counts ranging from 1.41 to 5.68 log_10_ CFU per nipple (Table 2).

#### Detection of *Salmonella*

In NCDE installed for one month, 7.14% (3 out of 42) of the samples were positive for *Salmonella*. One P2 sample and two samples among P3 to P6 from two different drinkers harboured *Salmonella*. All three *Salmonella* isolates were serotyped as *S.* Typhimurium. In NCDE installed for three months, 4.76% (2 out of 42) of the samples were *Salmonella-*positive, i.e. one P2 sample and one of P3 to P6. Both isolates were *S.* Derby and they originated from the same drinker.

#### Detection of pathogenic *Yersinia enterocolitica*

No pathogenic *Yersinia enterocolitica* was detected in the NCDE installed for one month. In NCDE installed for three months, 7.14% (3 out of 42) of the samples were positive, i.e. one P2 sample and two of P3 to P6, originating from two different drinkers.

#### Detection of *Listeria monocytogenes*

*Listeria monocytogenes* only occurred in the NCDE installed for three months, respectively once in a P2 sample and once in a P3 to P6 sample, so 4.76% (2 out of 42) of samples from one drinker harboured this pathogen.

### Detection of MRSA

MRSA was not detected in the NCDE installed for one month, whereas in NCDE installed for three months, MRSA occurred in one P2 sample and in two P3 to P6 samples, so 7.14% (3 out of 42) of samples from three different drinkers contained this pathogen.

#### Comparison of NCDE and UCDE

All results of the tested bacterial counts or the presence of specific microorganisms on the NCDE were compared and examined for statistical significance between the two tested time durations the equipment was used in the abattoir. Furthermore, the results of the NCDE were compared to the results of UCDE tested in the previous study (Buder et al. 2023) for the same installation periods. The different locations of Drinkers A–F in the lairage showed no clear influence on the bacterial loads detected in the UCDE (Buder et al. 2023) or in the NCDE (this study), and therefore, these results are not further described.

### APC

**Fig. 3:**
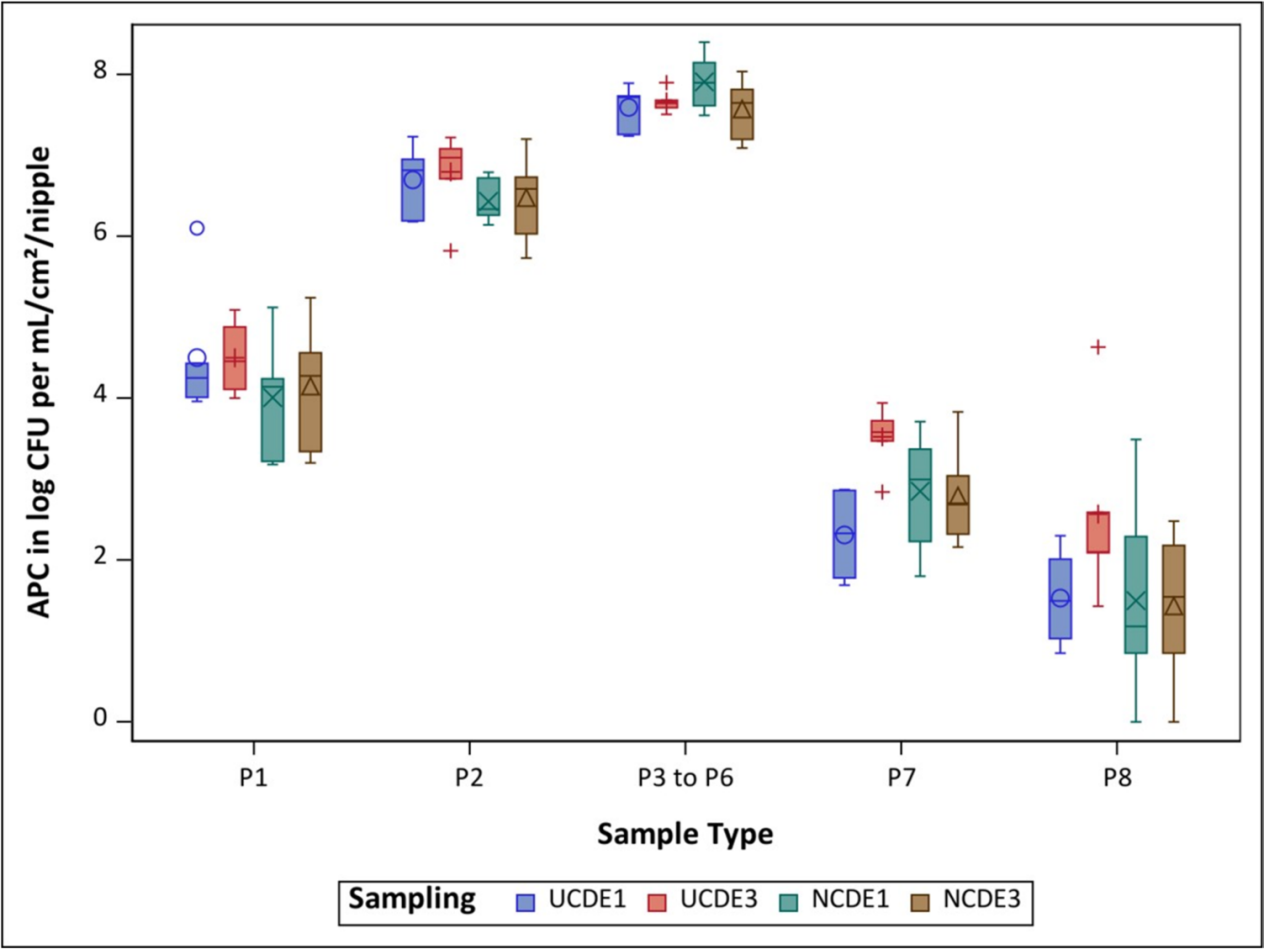
Overview of the mean total aerobic plate count (APC) for all six drinkers per sample type and sampling for nano-coated drinking equipment installed for one (NCDE1 - ) and three (NCDE3 - ) months and for uncoated drinking equipment installed for one (UCDE1 - +) and three (UCDE3 - ○) months (Buder et al. 2023); CFU – colony forming units; P1 – water sample in log CFU/ per mL; P2 – pooled nipple swab sample in log CFU/ per cm^2^; P3 to P6 – individual nipple sample in log CFU/ per nipple; P7 – pooled lower pipe section swab sample in log CFU/ per cm^2^; P8 – pooled upper pipe section swab sample in log CFU/ per cm^2^

Comparing the NCDE installed for one and three months in regard to APC, both samplings showed bacterial growth in 97.9% (47 out of 48) of the samples. The only sample type in both samplings (for the 1- and 3-month durations) that did not produce any bacterial growth were samples of P8. In detailed comparisons of the individual sample types, the NCDE installed for three months showed higher mean APCs in P1 and P2, while NCDE installed for one month had higher mean APCs in P3 to P6, P7 and P8 (Fig. 3). None of these findings showed a statistically significant difference between the two time durations for which the NCDE was installed (Table 3).

Comparing the APCs related to NCDE installed for one month and UCDE installed for one month, P1, P2 and P8 showed a higher mean APC in UCDE (Buder et al. 2023), while P3 to P6 and P7 showed a higher mean APC in NCDE. Overall, UCDE installed for one month showed bacterial growth in 100% (48 out of 48) of the samples. There was no statistically significant difference between the APC findings in NCDE and UCDE after one month of use. Comparing the samplings of NCDE and UCDE, both installed for three months, all mean APCs were numerically higher in the UCDE, and 97.9% (47 out of 48) of the UCDE samples showed bacterial growth (Buder et al. 2023), but no statistically significant differences compared with APC findings for NCDE were found (Fig. 3, Table 3).

#### EB

**Fig. 4:**
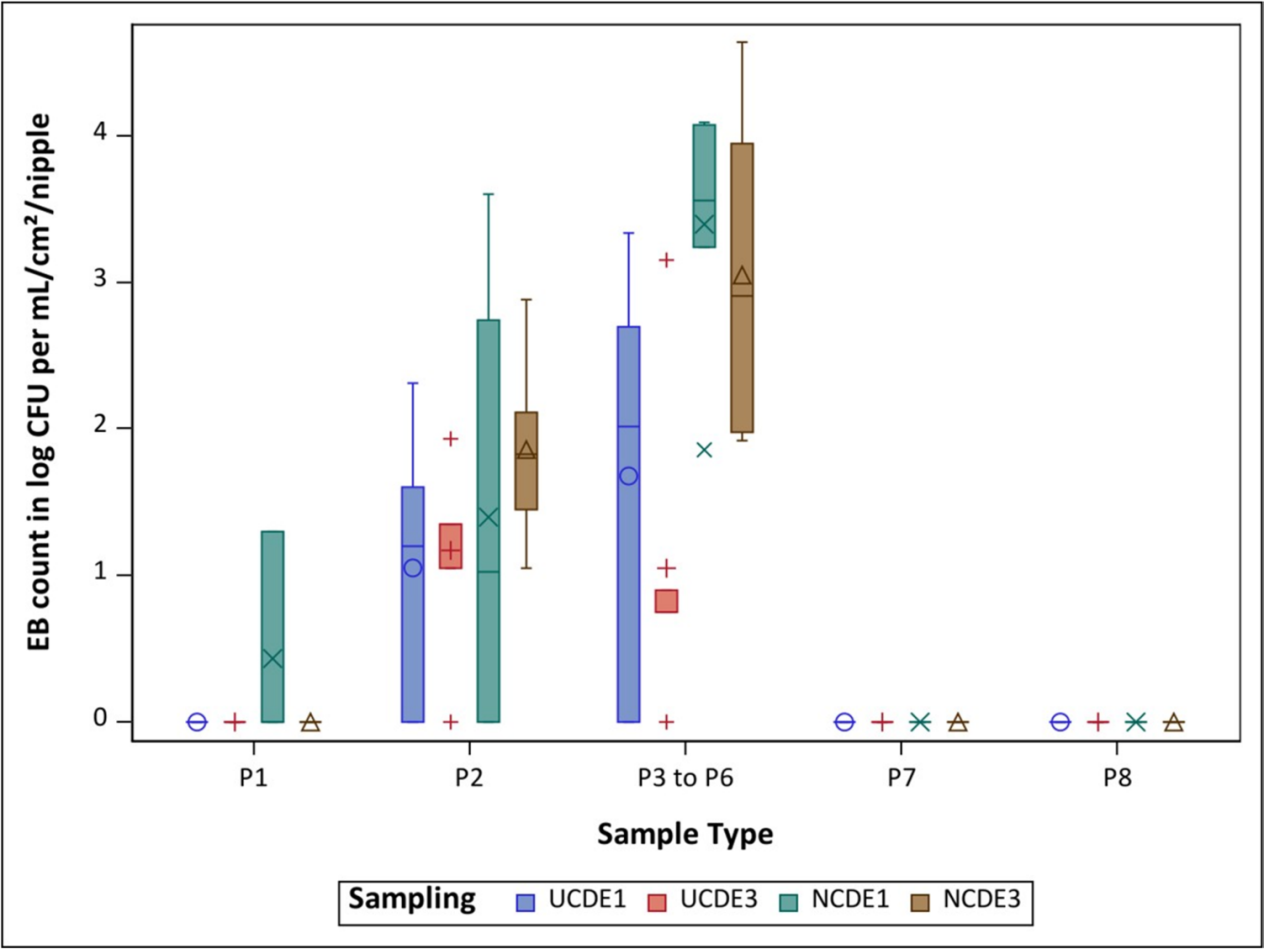
Overview of the mean value for all six drinkers per sample type and sampling for *Enterobacteriaceae *(EB) counts for nano-coated drinking equipment installed for one (NCDE1 - ) and three (NCDE3 - ) months and uncoated drinking equipment installed for one (UCDE1 - +) and three (UCDE3 - ○) months (Buder et al. 2023); CFU – colony forming units; P1 – water sample in log CFU/ per mL; P2 – pooled nipple swab sample in log CFU/ per cm^2^; P3 to P6 – individual nipple sample in log CFU/ per nipple; P7 – pooled lower pipe section swab sample in log CFU/ per cm^2^; P8 – pooled upper pipe section swab sample in log CFU/ per cm^2^

In NCDE installed for one month, 54.2% (26 out of 48) of the samples harboured EB, whereas in NCDE installed for three months, 50% (24 out of 48) of the samples were positive for EB. While in NCDE installed for one month, two samples of P1 were positive for EB, EB were not detected in the P1 samples for NCDE installed for three months. In P2 samples, EB were found more frequently in the NCDE installed for three months, with positive findings in all six drinkers, while in the NCDE installed for one month, EB were detected in three drinkers. NCDE installed for one month showed a higher mean EB count for P3 to P6 samples than NCDE installed for three months. In the sample types P7 and P8, no EB were detected in any of the samplings (Fig. 4). There was no statistically significant difference in the EB findings between the NCDE installed for one month and three months (Table 3).

When comparing the NCDE installed for one month with the UCDE installed for one month, higher mean EB counts were found in all positive sample types for the NCDE. Additionally, the UCDE installed for one month had 31.3% (15 out of 48) of positive samples (Buder et al. 2023). The NCDE installed for three months and the UCDE installed for three months showed similar tendencies. All positive sample types had higher mean EB counts in the NCDE compared to in the UCDE (Buder et al. 2023) (Fig. 4), which harboured EB in 27.1% (13 out of 48) of samples. No statistically significant differences in mean EB counts between the two installation durations or the coating status were measured (Table 3).

#### *Pseudomonas* spp. counts

**Fig. 5:**
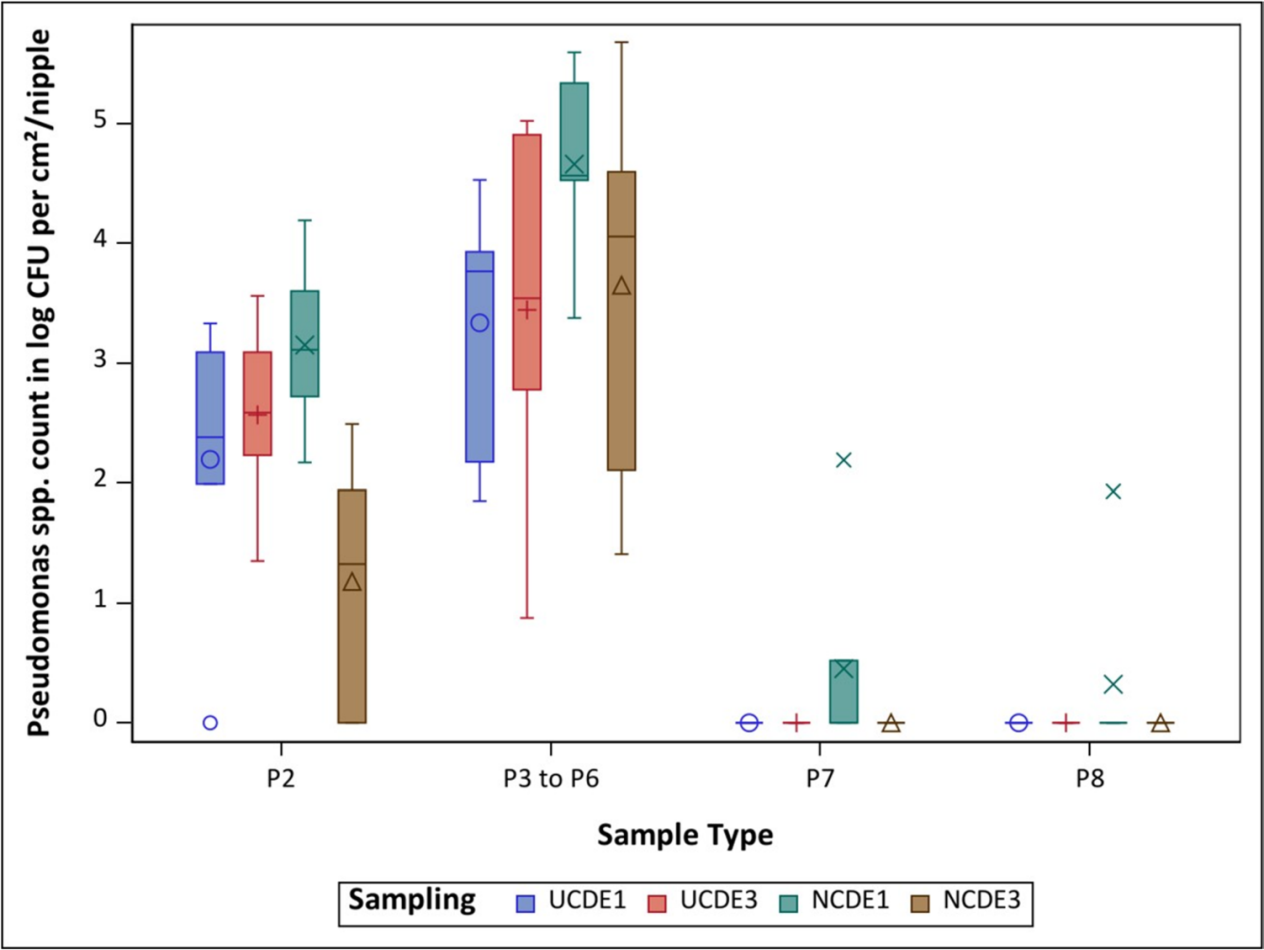
Overview of the mean value for all six drinkers per sample type and sampling for *Pseudomonas *spp. for nano-coated drinking equipment installed for one (NCDE1 - ) and three (NCDE3 - ) months and uncoated drinking equipment installed for one (UCDE1 - +) and three (UCDE3 - ○) months (Buder et al. 2023); CFU – colony forming units; P2 – pooled nipple swab sample in log CFU/ per cm^2^; P3 to P6 – individual nipple sample in log CFU/ per nipple; P7 – pooled lower pipe section swab sample in log CFU/ per cm^2^; P8 – pooled upper pipe section swab sample in log CFU/ per cm^2^

In total, *Pseudomonas* spp. was detected in 76.2% (32 out of 42) of the samples in the NCDE installed for one month. In comparison, 52.4% (22 out of 42) of the samples were positive for *Pseudomonas* spp. in NCDE installed for three months. Furthermore, the mean *Pseudomonas* spp. counts of sample types P2 and P3 to P6 were higher in NCDE installed for one month than in the same samples from equipment installed for three months. In the NCDE installed for three months, *Pseudomonas* spp. was not detected in P7 or P8, while in NCDE installed for one month, *Pseudomonas* spp. was found in these sample types. In the previous study, *Pseudomonas* spp. was not found in P7 or P8 samples from the UCDE (Buder et al. 2023). Therefore, among all P7 and P8 samples, *Pseudomonas* spp. was detected only in NCDE installed for one month (Fig. 5). A statistically significant difference was observed in *Pseudomonas* spp. counts in P2 samples from the NCDE installed for one and three months (*P* = 0.0324, Table 3).

Comparing NCDE and UCDE installed for one month, NCDE showed higher mean *Pseudomonas* spp. counts in P2 and P3 to P6 than the UCDE (Buder et al. 2023). NCDE and UCDE installed for three months were slightly different with respect to *Pseudomonas* spp. counts. While UCDE showed a higher mean *Pseudomonas* spp. count in P2 (Buder et al. 2023), NCDE showed a higher mean *Pseudomonas* spp. count in P3 to P6 (Fig. 5). Overall, 57.1% (24 out of 42) of the samples from UCDE installed for one month and 59.5% (25 out of 42) of the samples from UCDE installed for three months were positive for *Pseudomonas* spp. (Buder et al. 2023). There was no statistically significant difference in the occurrence of *Pseudomonas* spp. between the NCDE and the UCDE (Table 3).

#### Salmonella

*Salmonella* was detected in 7.1% of the samples (3 out of 42) in NCDE installed for one month and in 4.8% of the samples (2 out of 42) in NCDE installed for three months. In comparison, in the previous related study, in UCDE installed for one month, 9.5% of the samples (4 out of 42) and in UCDE installed for three months, 2.4% of the samples (1 out of 42) were positive for *Salmonella* (Buder et al. 2023). Considering the previous and this current study, *Salmonella* were isolated from some P2 samples, with one positive sample in each sampling, except for the P2 samples of UCDE installed for three months, from which *Salmonella* was not isolated. All other *Salmonella*-positive samples were type P3 to P6, with two in NCDE installed for one month, three in UCDE installed for one month (Buder et al. 2023) and respectively one each in NCDE [this study] and UCDE (Buder et al. 2023) installed for three months (Table 1). *S.* Typhimurium was isolated from NCDE installed for one month and UCDE installed for three months (Buder et al. 2023). *S.* Derby was isolated from UCDE installed for one month (Buder et al. 2023) and NCDE installed for three months.

**Table 1:** Overview of *Salmonella*, pathogenic *Y. enterocolitica*, *L. monocytogenes* and MRSA in the tested sample types and samplings for nano-coated drinking equipment installed for one (NCDE1) and three (NCDE3) months and uncoated drinking equipment installed for one (UCDE1) and three (UCDE3) months (Buder et al. 2023)

Pathogen	Sample type	Sampling			
		**NCDE1**	**NCDE3**	**UCDE1**	**UCDE3**
*Salmonella*	P2	+	+	+	−
	P3 to P6	+ +	+	+ + +	+
	P7	−	−	−	−
	P8	−	−	−	−
Pathogenic *Y. enterocolitica*	P2	−	+	+	+
	P3 to P6	−	+ +	+ +	−
	P7	−	−	−	−
	P8	−	−	−	−
*L. monocytogenes*	P2	−	+	−	−
	P3 to P6	−	+	−	−
	P7	−	−	−	−
	P8	−	−	−	−
MRSA	P2	−	+	−	−
	P3 to P6	−	+ +	+ + +	−
	P7	−	−	−	−
	P8	−	−	−	−

P2—pooled nipple swab sample; P3 to P6—individual nipple sample; P7—pooled lower pipe section swab sample; P8—pooled upper pipe section swab sample; − indicates samples which had no positive finding for the specific pathogen; + indicates the samples which had one positive finding for the specific pathogen; +  + indicates the samples which had two positive findings for the specific pathogen; +  +  + indicates the samples which had three positive findings for the specific pathogen.

#### Pathogenic *Y. enterocolitica*

Pathogenic *Y. enterocolitica* was not detected in NCDE installed for one month but was detected in 7.1% (3 out of 42) of the samples of the UCDE in use in the abattoir lairage for the same duration (sample types P2 and P3 to P6) (Buder et al. 2023). Similarly, *Y. enterocolitica* was detected in the NCDE installed for three months in 7.1% (3 out of 42) of the samples (sample types P2 and P3 to P6), while in the UCDE installed for three months 2.4% of the samples (P2; 1 out of 42) harboured this pathogen (Buder et al. 2023) (Table 1).

#### L. monocytogenes

*L. monocytogenes* was isolated only from the NCDE installed for three months, distributed over sample types P2 and P3 to P6, with 4.8% (2 out of 42) of samples being positive. *L. monocytogenes* was not detected in UCDE, regardless of the duration of installation (Buder et al. 2023), or in NCDE installed for one month (Table 1).

### MRSA

MRSA was not detected in samples from NCDE installed for one month or in UCDE installed for three months (Buder et al. 2023). Regarding NCDE installed for three months and UCDE installed for one month, 7.1% (3 out of 42) of the respective samples were positive for MRSA. This microorganism was detected in sample types P2 and P3 to P6 for NCDE installed for three months and in sample type P3 to P6 for UCDE installed for three months (Buder et al. 2023) (Table 1).

#### SEM–EDX analysis

**Fig. 6:**
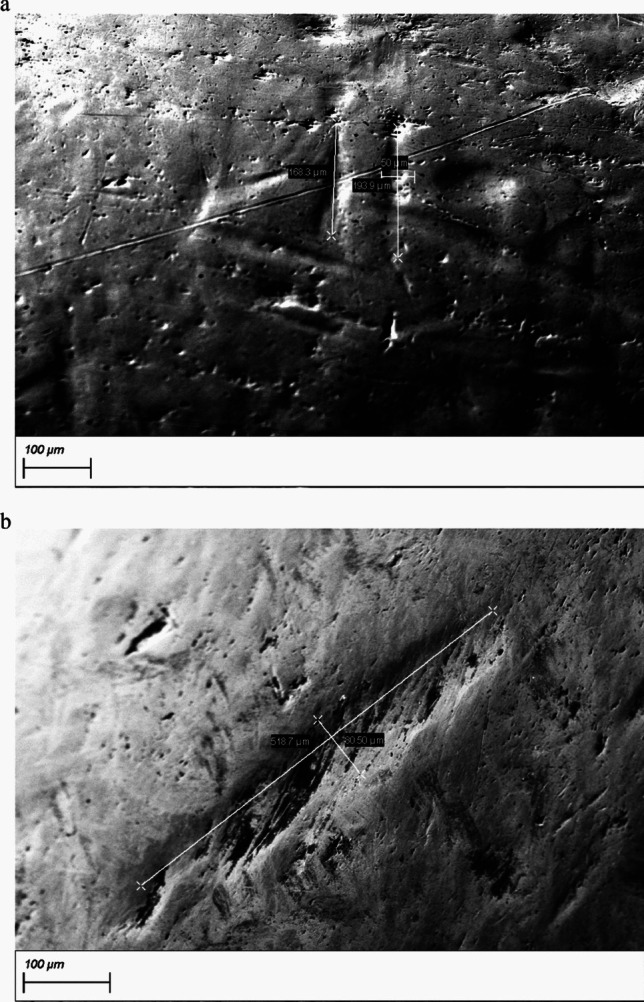
Scanning electron microscope (SEM) images of the anterior parts of nipples from the nano-coated drinking equipment (**a**) and from the uncoated drinking equipment (**b**). Length and width in µm are indicated for the notches detected on the surfaces of the nipples

The surface properties of the disassembled nipples of NCDE and UCDE installed for three months were examined using SEM and revealed the presence of multiple indentations and notches on the anterior surfaces (Fig. 6), but not on the posterior surfaces of the nipples.

**Fig. 7:**
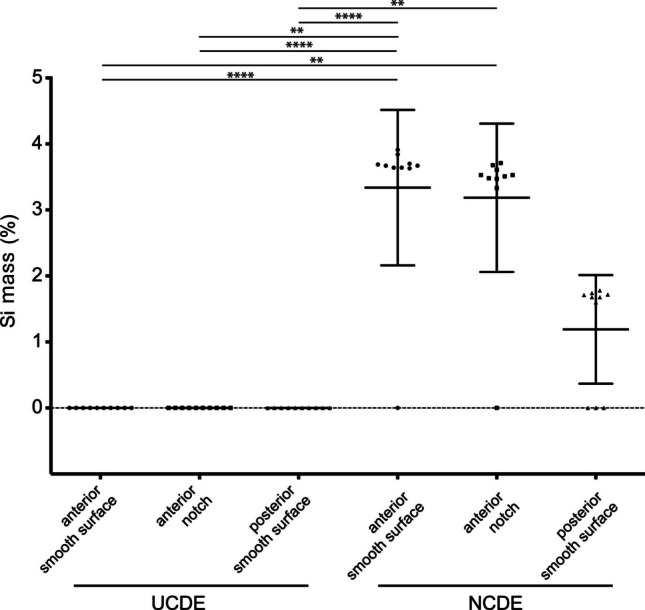
Relative mass of silicon (Si) in percentage (%) within the smooth regions, i.e., smooth surface of anterior and posterior regions, and within notches of anterior regions, on a nipple from the uncoated drinking equipment (UCDE) and a nipple from the nano-coated drinking equipment (NCDE). Silicon was detected by energy dispersive x-ray (EDX) analysis. Data represent mean ± standard deviation (SD). Ten different spots were analysed for each region and nipple. Solid lines indicate a significant difference in the relative Si mass between the distinct regions analysed with ** - *P *< 0.01, **** - *P *< 0.0001

The EDX analysis showed that in all surface regions of the nipple of NCDE (i.e. smooth surface of the anterior and posterior nipple region, notch of the anterior nipple region), iron (Fe) was the major element detected (61% in the anterior and posterior position and 58% in the notch). In addition, manganese (Mn; 20% in all positions), chrome (Cr; 17% in the anterior position, 18% in the posterior position and 19% in the notch) and a small amount of silicon (Si; 2% in the anterior position, 1% in the posterior position and 3% in the notch) were detected in all measurements. Mean relative amounts of Si were higher in the anterior regions than in the posterior region of the nipple from the NCDE; however, these differences were not statistically significant (Fig. 7).

Furthermore, minor amounts (< 1%) of the following elements were detected in individual measurements on the surface of the nipple from the NCDE: tungsten (W), tantalum (Ta), calcium (Ca), ytterbium (Yb), osmium (Os), copper (Cu), phosphorus (P) and platinum (Pt), which are considered by the authors to represent measurement errors (data not shown).

Similar to the nipple from the NCDE, on all surface regions of the nipple from the UCDE (i.e. smooth surfaces of the anterior and posterior nipple regions, notches of the anterior nipple region), Fe was the main element detected (100% in the anterior and posterior position and 87% in the notch), besides smaller amounts of other metals, e.g. cobalt (Co; 13% in the notch). Si was not detected on the surface regions of the nipple from the UCDE. When comparing the nipples of the NCDE and UCDE for the relative amounts of Si measured on the surface regions, significant differences (*P* < 0.01; Fig. 7) were found between the anterior surface regions of the nipple of NCDE (i.e. between the smooth surfaces and notches of the anterior nipple region) and between all surface regions of the nipple of UCDE (i.e. smooth surfaces of the anterior and posterior nipple region, notches of the anterior nipple region) (Fig. 7).

## Discussion

### Study design

In this study, the results regarding nano-coated nipple drinkers installed in a pig abattoir lairage for different durations (one and three months) were compared with the results of a previously conducted study, which focused on the general occurrence of biofilm and specific pathogens on uncoated drinkers in the same pig abattoir lairage (Buder et al. 2023). It was essential to ensure comparability between these four drinker samplings, which is why the same number/type of drinkers and samples, and the same sampling techniques were used. Additionally, the lairage pens needed to stay comparable over time, while sampling all lairage pens was not possible at the time. Nevertheless, given the high variability of the data and the presence of outliers, a larger sample size would have been desirable to limit possible misinterpretation of the effects. Further studies should include a bigger sample size to ensure the reliability of the results.

### Influence of nano-coating on bacterial load and occurrence of specific pathogens

The aim of this study was to evaluate whether a nano-coating of silicon dioxide was able to indirectly reduce the bacterial load on the surfaces of the drinking equipment used in lairage pens of pig abattoirs. The specific silicon dioxide nano-coating applied did not statistically reduce the bacterial load or specific pathogens on the coated stainless-steel drinkers compared to the UCDE (Buder et al. 2023) used under the same conditions and for the same time durations. The only statistically significant difference was in *Pseudomonas* spp. counts in pooled swab samples from all four nipple drinkers per drinker, with regard to NCDE installed for one month and NCDE installed for three months. The *Pseudomonas* spp. counts in NCDE installed for three months were statistically significantly lower than in NCDE installed for one month. This could mean that in the nano-coated drinkers, the lower amount of *Pseudomonas* spp., regarded as a strong biofilm builder (Sauer et al. 2002), might have reduced the general amount of biofilm mass. However, the other not statistically significant results demonstrate that no reduction effect occurred for the other bacteria examined, and the *Pseudomonas* spp. reduction might be coincidental. Simultaneously to this study, other recent studies did not find any statistically significant decrease in the occurrence of specific pathogens, such as *L. monocytogenes* (Hillig et al. 2023) or *Salmonella* (Blaeske et al. 2024), on silicon dioxide nano-coated stainless steel. This stands in contrast to prior research in different environments, including stainless steel under aquatic influences, which demonstrated a statistically significant reduction of 3 log levels of biofilm on surfaces coated with nano silica after one month (Turetgen 2020). In the current study, the stainless-steel surfaces of the used nipple drinkers were examined with electron microscopy and showed a high occurrence of notches. These notches were only detected on the anterior, but not in the posterior surface regions of the nipples removed from the NCDE and UCDE. Given that only the posterior region of nipples is covered by a cone (Fig. 2) and, thus, is protected from the pigs’ teeth, it can be assumed that most of the notches observed in the anterior region originated from the pigs’ teeth contacting the stainless steel during drinking or playing processes. This, in addition to possible material failures, might have influenced the current results.

Using EDX analysis, Si was only found on the nipple surface of the NCDE and not on that of the UCDE, indicating that the nano-coating with silicon dioxide was successfully applied. Si was detected throughout the nipple surface, including the depth, the margins and the outside of the notches of the NCDE. Thus, on used NCDE, the nano-coating was resistant to focal points of high pressure or stress induced by the pigs’ teeth while biting on the nipple drinker. However, the coating was unable to sufficiently prevent the adherence, enhance the detachment or decrease the reproduction of biofilm-producing bacteria. Other coating modifications might result in lesser biofilm formation.

One explanation for the silicon dioxide nano-coating not effecting desirable bacterial reductions could be the rougher surface of the stainless steel used in the abattoir industry compared to stainless steel used in the food industry (e.g. during packaging, processing) or the medical sector, where nano-coating is used successfully (Ferraris et al. 2017; Pérez et al. 2022). This theory has been refuted by Di Cerbo et al. (2021), who proved that different levels of roughness of stainless-steel surfaces do not show a correlation with the adhesion of bacteria. However, they noted that sanitary treatment of stainless steel, which produced the only statistically significant effect against bacterial contamination in their study, leads to corrosion due to the aggressive characteristics of effective products. This, in turn, could give bacteria more opportunities to adhere and distribute. They concluded that nano-coating could prevent corrosion and, therefore, reduce bacterial contamination in combination with sanitary treatments (Di Cerbo et al. 2021).

### Influence of seasonal change

Seasonal changes might have also made an impact on the biofilm formation (Schmidt et al. 2016), but it is unknown to what extent. Temperatures were only measured inside and outside the lairage on the day of sampling, and the thorough analysis (not shown or discussed) showed temperature was not linked with the bacterial findings of this or the previous (Buder et al. 2023) study. Moreover, the average temperatures during the 1- or 3-month periods in our study are unknown, as are the meteorological changes during these periods. Another factor contributing to the seasonal influence is the amount of water flowing through the drinking equipment. While warmer temperatures generally result in more biofilm due to better growing conditions for bacteria (Diaz Villanueva et al. 2011), these conditions also lead to increased water flow because the pigs drink more water on warmer days, which disturbs biofilm development due to higher sheer pressure (Olczak et al. 2015).

To ensure better comparability, different coatings and different time durations should be examined simultaneously. This was not the case in this study, because the main focus was to compare the nano-coating on the exact same type of drinkers installed for the same durations as we used previously (Buder et al. 2023).

### Possible sources of bacterial contamination

Bacterial contamination was detected in the water samples, but the main water distribution system can be excluded as a possible source for much of the bacterial contamination because the water used by the abattoir is tested regularly and is within the sanitary standards for drinking water in Germany (TrinkwV 2023). Notably, the pigs’ mouths are not usually clean, which resulted in visible dirt on the nipple drinkers at sampling. To ensure the authenticity of the water samples, the water was released and collected in the sample tube without the drinker being cleaned before sampling, because other pigs would be exposed to the same conditions. Any cleaning of the nipple drinkers before sampling would have potentially modified the results. In addition, the distribution of positive samples, especially in terms of general bacterial growth (APC), illustrates the retrograde growth of biofilm from the exposed nipples up to the end of the drinkers (P8). This proves the biofilm is able to grow against the water flow at least 75 cm, because that was the minimum height at which we sampled the upper pipe section.

Even though *L. monocytogenes* was detected for the first time herein, but not in the previous study in the same environment (Buder et al. 2023), these findings must be considered in the context of biofilm and *L. monocytogenes* dynamics. Infected pigs using the drinking equipment on the sampling day could have transmitted *L. monocytogenes* from their infected tonsils (Autio et al. 2004) on to the drinkers, while bacteria could have occasionally detached from the biofilm cluster, so both these factors could have influenced this finding (Banerjee et al. 2019). Furthermore, it cannot be guaranteed that the drinking equipment in the previous study (Buder et al. 2023) did not contain *L*. *monocytogenes*, despite this pathogen not being detected. This could be due to the fact that some bacteria, including *L. monocytogenes*(Gião and Keevil 2014), are able to enter a viable but non-culturable state. If such bacteria occur in any tested environment, they cannot be isolated by culture methods (Byrd et al. 1991; Guo et al. 2021; Wingender and Flemming 2011), such as were used in this and the previous study. Additionally, the assumption that *L. monocytogenes* was harboured on the drinking equipment because it was coated with silicon dioxide nano-coating cannot be made. Similar assumptions cannot be made for the other specific pathogens either.

Finally, pigs function as a reservoir for some specific pathogens (Deane et al. 2022; Lewis et al. 2008; Raymond et al. 2019; Schoder et al. 2022). The bacteria can be isolated from different pig organs, notably for the purposes of this study, from the tonsils (Autio et al. 2004; Jouy et al. 2012; Nesbakken et al. 2003; Oswaldi et al. 2021; Swanenburg et al. 2001), and are likely able to be transmitted onto the drinking equipment and via the drinkers onto/into uninfected pigs. This would, subsequently, lead to freshly infected pigs entering the slaughter line, where the bacteria could spread by cross-contamination. This study proves that using drinkers with the specific silicon dioxide nano-coating applied did not reduce the bacterial loads or the occurrence of specific pathogens in/on the abattoir lairage drinkers with respect to uncoated drinkers. From that, it can be assumed that on nano-coated drinkers, there remain sufficient quantities of undesirable bacteria to cause pathogen transfer or pig infection via the drinking equipment in the lairage pens prior to slaughter.

## Conclusion

As mentioned above, many studies showed the usefulness of nano-coatings in different fields. At the time of publication, this was the first study to investigate, in pig abattoir lairage pens, the occurrence of specific pathogens on/in drinking equipment coated with silicon dioxide in comparison to uncoated drinking equipment in the same pig lairage pens. No statistically significant reductions in the levels of APC or EB or in the occurrences of *Salmonella*, *Y. enterocolitica* or pathogenic MRSA were detected. *L. monocytogenes* was detected for the first time in the examined pig lairage drinking equipment. A statistically significant reduction of *Pseudomonas* spp. was detected only once, which, in comparison to the other findings, only pertains to a very small part of this study and does not prove a reliable reduction of bacterial contamination in general. Therefore, the specific silicon dioxide nano-coating applied cannot be recommended for this field of application at this time. Despite the results of this study, other combinations of methods, e.g. sanitary treatments along with nano-coating, in addition to differently modified surfaces with other nano-coated substances, might be applied to increase the effectiveness and should be investigated in further studies.

## Data Availability

The datasets generated during and/or analysed during the current study are available from the corresponding author on reasonable request.

## Appendix 1

**Table 2:** Descriptive statistics for total aerobic plate count (APC), *Enterobacteriaceae* (EB) and *Pseudomonas* spp. (PS) by sample type and sampling and the results for Kruskal–Wallis test and Dwass-Steel-Critchlow-Fligner post hoc test

Microorganism	Sample type	Sampling	*N*	Mean	Median	STD	CV	Min	P5	P95	Max	Missing
APC	P1	UCDE1	6	4.50	4.25	0.80	17.85	3.96	3.96	6.10	6.10	0
		UCDE3	6	4.50	4.46	0.42	9.43	4.00	4.00	5.09	5.09	0
		NCDE1	6	4.01	4.14	0.73	18.11	3.18	3.18	5.12	5.12	0
		NCDE3	6	4.15	4.28	0.77	18.64	3.20	3.20	5.24	5.24	0
	P2	UCDE1	6	6.70	6.82	0.43	6.45	6.18	6.18	7.23	7.23	0
		UCDE3	6	6.80	6.97	0.51	7.46	5.82	5.82	7.22	7.22	0
		NCDE1	6	6.43	6.34	0.27	4.15	6.14	6.14	6.79	6.79	0
		NCDE3	6	6.48	6.59	0.53	8.12	5.73	5.73	7.20	7.20	0
	P3–P6	UCDE1	6	7.59	7.72	0.27	3.62	7.24	7.24	7.89	7.89	0
		UCDE3	6	7.66	7.64	0.13	1.72	7.51	7.51	7.90	7.90	0
		NCDE1	6	7.91	7.90	0.36	4.51	7.49	7.49	8.40	8.40	0
		NCDE3	6	7.57	7.65	0.38	4.97	7.09	7.09	8.04	8.04	0
	P7^a^	UCDE1	6	2.31	2.33	0.51	21.98	1.69	1.69	2.87	2.87	0
		UCDE3	6	3.52	3.58	0.37	10.56	2.84	2.84	3.94	3.94	0
		NCDE1	6	2.85	3.00	0.75	26.22	1.80	1.80	3.71	3.71	0
		NCDE3	6	2.79	2.70	0.60	21.38	2.16	2.16	3.83	3.83	0
	P8	UCDE1	6	1.53	1.50	0.56	36.28	0.85	0.85	2.30	2.30	0
		UCDE3	5	2.57	2.10	1.22	47.67	1.43	1.43	4.63	4.63	1
		NCDE1	6	1.50	1.18	1.23	81.78	0.00	0.00	3.49	3.49	0
		NCDE3	6	1.43	1.55	0.90	63.08	0.00	0.00	2.48	2.48	0
EB	P1	UCDE1	6	0.00	0.00	0.00		0.00	0.00	0.00	0.00	0
		UCDE3	6	0.00	0.00	0.00		0.00	0.00	0.00	0.00	0
		NCDE1	6	0.43	0.00	0.67	154.92	0.00	0.00	1.30	1.30	0
		NCDE3	6	0.00	0.00	0.00		0.00	0.00	0.00	0.00	0
	P2	UCDE1	6	1.05	1.20	0.91	86.99	0.00	0.00	2.31	2.31	0
		UCDE3	6	1.17	1.35	0.64	54.76	0.00	0.00	1.93	1.93	0
		NCDE1	6	1.40	1.03	1.61	115.04	0.00	0.00	3.60	3.60	0
		NCDE3	6	1.86	1.83	0.63	33.77	1.05	1.05	2.88	2.88	0
	P3–P6^a^	UCDE1	6	1.68	2.01	1.41	84.14	0.00	0.00	3.34	3.34	0
		UCDE3	6	1.05	0.75	1.08	102.62	0.00	0.00	3.15	3.15	0
		NCDE1	6	3.39	3.56	0.82	24.30	1.86	1.86	4.09	4.09	0
		NCDE3	6	3.05	2.91	1.19	38.90	1.92	1.92	4.64	4.64	0
	P7	UCDE1	6	0.00	0.00	0.00		0.00	0.00	0.00	0.00	0
		UCDE3	6	0.00	0.00	0.00		0.00	0.00	0.00	0.00	0
		NCDE1	6	0.00	0.00	0.00		0.00	0.00	0.00	0.00	0
		NCDE3	6	0.00	0.00	0.00		0.00	0.00	0.00	0.00	0
	P8	UCDE1	6	0.00	0.00	0.00		0.00	0.00	0.00	0.00	0
		UCDE3	6	0.00	0.00	0.00		0.00	0.00	0.00	0.00	0
		NCDE1	6	0.00	0.00	0.00		0.00	0.00	0.00	0.00	0
		NCDE3	6	0.00	0.00	0.00		0.00	0.00	0.00	0.00	0
PS	P2^a^	UCDE1	6	2.20	2.38	1.21	55.02	0.00	0.00	3.33	3.33	0
		UCDE3	6	2.57	2.59	0.78	30.32	1.35	1.35	3.56	3.56	0
		NCDE1^b^	6	3.15	3.11	0.72	22.90	2.17	2.17	4.19	4.19	0
		NCDE3^b^	6	1.18	1.33	1.08	91.13	0.00	0.00	2.49	2.49	0
	P3–P6	UCDE1	6	3.34	3.77	1.07	32.00	1.85	1.85	4.53	4.53	0
		UCDE3	6	3.44	3.54	1.58	46.02	0.88	0.88	5.02	5.02	0
		NCDE1	6	4.66	4.56	0.78	16.66	3.38	3.38	5.59	5.59	0
		NCDE3	6	3.65	4.06	1.60	43.91	1.41	1.41	5.68	5.68	0
	P7	UCDE1	6	0.00	0.00	0.00		0.00	0.00	0.00	0.00	0
		UCDE3	6	0.00	0.00	0.00		0.00	0.00	0.00	0.00	0
		NCDE1	6	0.45	0.00	0.88	194.09	0.00	0.00	2.19	2.19	0
		NCDE3	6	0.00	0.00	0.00		0.00	0.00	0.00	0.00	0
	P8	UCDE1	6	0.00	0.00	0.00		0.00	0.00	0.00	0.00	0
		UCDE3	6	0.00	0.00	0.00		0.00	0.00	0.00	0.00	0
		NCDE1	6	0.32	0.00	0.79	244.95	0.00	0.00	1.93	1.93	0
		NCDE3	6	0.00	0.00	0.00		0.00	0.00	0.00	0.00	0

APC—total aerobic plate count; EB—Enterobacteriaceae count; PS—Pseudomonas spp. count; P1—mean value of the water samples in log CFU per mL; P2—mean value of the pooled swab samples from all four nipple drinkers in log CFU per cm^2^; P3–P6—mean value of the individual nipple samples in log CFU per nipple; P7—mean value of the pooled swab samples of the lower pipe section in log CFU per cm^2^; P8—mean value of the pooled swab samples of the upper pipe section in log CFU per cm^2^; N—sample size; STD—standard deviation; CV—coefficient of variation; Min—minimum; P5—5% quantile; P95—95% quantile; Max—maximum;.—no value; ^a^ Statistically significant effect (P < 0.05, Kruskal–Wallis test); ^b^ Statistically significant difference between the two samplings (P < 0.05, Dwass-Steel-Critchlow-Fligner post hoc test).

**Table 3:** Results of Kruskal–Wallis (KW) and Dwass-Steel-Critchlow-Fligner post hoc test to evaluate the pairwise differences of the bacterial counts between samplings by sample type for nano-coated drinking equipment installed for one (NCDE1) and three (NCDE3) months and uncoated drinking equipment installed for one (UCDE1) and three (UCDE3) months. The relationships between UCDE1 and UCDE3 have already been analysed in detail (Buder et al. 2023)

Microorganism	Sample type	KW statistic	DF	p1	Comparison	Wilcoxon Z	DSCF value	p2
APC	P1	1.6023633	3	0.6589	UCDE1 vs. NCDE1	0.9608	1.3587	0.7717
					UCDE3 vs. NCDE3	0.6405	0.9058	0.9189
					NCDE1 vs. NCDE3	− 0.5614	0.7940	0.9434
	P2	3.2680876	3	0.3521	UCDE1 vs. NCDE1	0.9625	1.3611	0.7708
					UCDE3 vs. NCDE3	1.2810	1.8116	0.5750
					NCDE1 vs. NCDE3	− 0.1604	0.2269	0.9985
	P3 to P6	2.2333333	3	0.5254	UCDE1 vs. NCDE1	− 0.9608	1.3587	0.7717
					UCDE3 vs. NCDE3	0.3203	0.4529	0.9886
					NCDE1 vs. NCDE3	1.4412	2.0381	0.4736
	P7	8.6733333	3	**0.0340**	UCDE1 vs. NCDE1	− 1.2810	1.8116	0.5750
					UCDE3 vs. NCDE3	1.9215	2.7175	0.2189
					NCDE1 vs. NCDE3	0.0000	0.0000	1.0000
	P8	3.2983639	3	0.3479	UCDE1 vs. NCDE1	0.4821	0.6818	0.9631
					UCDE3 vs. NCDE3	1.2780	1.8074	0.5770
					NCDE1 vs. NCDE3	− 0.1607	0.2273	0.9985
EB	P1	6.2727273	3	0.0991	UCDE1 vs. NCDE1	− 1.4832	2.0976	0.4476
					UCDE3 vs. NCDE3	0.0000	0.0000	1.0000
					NCDE1 vs. NCDE3	1.4832	2.0976	0.4476
	P2	2.9852658	3	0.3939	UCDE1 vs. NCDE1	− 0.3321	0.4696	0.9874
					UCDE3 vs. NCDE3	− 1.8578	2.6273	0.2465
					NCDE1 vs. NCDE3	− 0.4838	0.6842	0.9627
	P3 to P6	10.737347	3	**0.0132**	UCDE1 vs. NCDE1	− 2.2457	3.1759	0.1111
					UCDE3 vs. NCDE3	− 2.4189	3.4208	0.0735
					NCDE1 vs. NCDE3	0.1601	0.2265	0.9985
	P7	0	3	1.0000	UCDE1 vs. NCDE1	0.0000	0.0000	1.0000
					UCDE3 vs. NCDE3	0.0000	0.0000	1.0000
					NCDE1 vs. NCDE3	0.0000	0.0000	1.0000
	P8	0	3	1.0000	UCDE1 vs. NCDE1	0.0000	0.0000	1.0000
					UCDE3 vs. NCDE3	0.0000	0.0000	1.0000
					NCDE1 vs. NCDE3	0.0000	0.0000	1.0000
PS	P2	9.6426289	3	**0.0219**	UCDE1 vs. NCDE1	− 1.6013	2.2646	0.3778
					UCDE3 vs. NCDE3	2.0853	2.9491	0.1578
					NCDE1 vs. NCDE3	2.7270	3.8565	**0.0324**
	P3 to P6	4.1133333	3	0.2495	UCDE1 vs. NCDE1	− 2.0817	2.9439	0.1591
					UCDE3 vs. NCDE3	− 0.1601	0.2265	0.9985
					NCDE1 vs. NCDE3	0.9608	1.3587	0.7717
	P7	6.2608696	3	0.0996	UCDE1 vs. NCDE1	− 1.4771	2.0889	0.4514
					UCDE3 vs. NCDE3	0.0000	0.0000	1.0000
					NCDE1 vs. NCDE3	1.4771	2.0889	0.4514
	P8	3	3	0.3916	UCDE1 vs. NCDE1	− 1.0000	1.4142	0.7494
					UCDE3 vs. NCDE3	0.0000	0.0000	1.0000
					NCDE1 vs. NCDE3	1.0000	1.4142	0.7494

APC—total aerobic plate count; EB—Enterobacteriaceae count; PS—Pseudomonas spp. count; P1—water sample in log CFU per mL; P2—pooled swab sample from all four nipple drinkers in log CFU per cm^2^; P3 to P6—mean value of the four individual nipple samples in log CFU per nipple; P7—pooled swab sample of the lower pipe section in log CFU per cm^2^; P8—pooled swab sample of the upper pipe section in log CFU per cm^2^; . —no value; DF—degrees of freedom; p1—P value for Kruskal–Wallis test; DSCF—Dwass-Steel-Critchlow-Fligner test; p2—P value for DSCF; significant P values are shown in bold-face type.
